# Surgery or No Surgery? Exploring the Dilemma of Epistaxis Management in Patients with HHT

**DOI:** 10.3390/jcm13061688

**Published:** 2024-03-15

**Authors:** Giulio Cesare Passali, Mariaconsiglia Santantonio, Nadia Vecchioli, Michela Sollazzo, Rolando Rolesi, Ilenia Marotta, Luigi Corina, Maria Elena Riccioni, Eleonora Gaetani, Jacopo Galli

**Affiliations:** 1 Università Cattolica del Sacro Cuore, Largo F. Vito, 00168 Rome, Italy; giuliocesare.passali@unicatt.it (G.C.P.); mariaelena.riccioni@policlinicogemelli.it (M.E.R.); eleonora.gaetani@policlinicogemelli.it (E.G.); jacopo.galli@policlinicogemelli.it (J.G.); 2 Division of Otorhinolaryngology, Fondazione Policlinico Universitario A. Gemelli IRCCS, Università Cattolica del Sacro Cuore, 00168 Rome, Italy; nadia.vecchioli01@icatt.it (N.V.); michela.sollazzo01@icatt.it (M.S.); rolando.rolesi@policlinicogemelli.it (R.R.); ilenia.marotta01@icatt.it (I.M.); luigi.corina@policlinicogemelli.it (L.C.); 3 Multidisciplinary Gemelli Group for HHT, Fondazione Policlinico Universitario A. Gemelli IRCCS, Università Cattolica del Sacro Cuore, 00168 Rome, Italy; 4 Ospedale Pediatrico Bambino Gesù, Via Torre di Palidoro s.n.c, 00054 Fiumicino, Italy; 5 Department of Medical and Surgical Sciences, Fondazione Policlinico Universitario A. Gemelli IRCCS, Università Cattolica del Sacro Cuore School of Medicine, 00168 Rome, Italy; 6 Department of Translational Medicine and Surgery, Fondazione Policlinico Universitario A. Gemelli IRCCS, Università Cattolica del Sacro Cuore, 00168 Rome, Italy

**Keywords:** hereditary hemorrhagic telangiectasia, rhinology, epistaxis

## Abstract

**Background**: Epistaxis, particularly in Hereditary Hemorrhagic Telangiectasia (HHT) patients, is a common otolaryngological emergency, often requiring complex management. A hierarchy of increasingly invasive interventions, from external compression of the nasal pyramid to nostril closure, is typically proposed and applied. **Methods**: We conducted a retrospective study on HHT patients to assess the effectiveness and longevity of invasive procedures postoperatively. Data were collected using the Epistaxis Severity Score (ESS) questionnaire. The primary focus was on changes in the frequency and intensity of epistaxis, while the secondary focus was on the overall quality of life. **Results**: This study found that invasive procedures initially improved the frequency and intensity of epistaxis in HHT patients. However, within 1 to 9 months postoperatively, these benefits often diminished, with hemorrhagic symptoms recurring at similar or worsened levels. **Conclusions**: The findings suggest a need for a cautious and restrained approach to using invasive treatments in managing epistaxis in HHT patients. Highly invasive procedures should be reserved for cases where less invasive methods fail, due to their temporary effectiveness and the risk of causing anatomical–functional changes in the rhino-sinus area, complicating future management of severe epistaxis.

## 1. Introduction

Hereditary Hemorrhagic Telangiectasia (HHT), also known as Rendu–Osler–Weber syndrome, is an autosomal dominant disorder characterized by abnormal vascular development and multiple arteriovenous malformations (AVMs) [1,2]. These AVMs can be small, like cutaneous or mucosal telangiectasias, or larger visceral malformations. The underlying pathology is a defect in the vascular wall [3]. The disorder is commonly manifested by spontaneous and recurrent nosebleeds (epistaxis), gastrointestinal bleeding, and pulmonary and cerebral arteriovenous malformations [4]. Approximately 1 in 5000 to 8000 individuals are affected by HHT, making it the second most common inherited bleeding disorder [5,6]. HHT belongs to the so-called orphan diseases being mainly treated in specialized centers [7,8]. The genetic basis of this condition involves changes in specific genes. These genes play a key role in regulating the signaling of the transforming growth factor (TGF)-β superfamily within the cells lining the blood vessels. Two main types of mutations have been identified: the first type affects the gene for endoglin, leading to HHT type 1, and the second type involves mutations in the activin receptor-like kinase (ALK1) gene, which results in HHT type 2. These mutations are detected using genetic testing techniques.

Management of epistaxis in HHT includes a range of medical and surgical interventions, based on the severity of epistaxis and the patient’s needs, particularly regarding compliance with medical therapy [9]. Surgical options like endoscopic surgery using argon plasma coagulation, laser, and quantum molecular resonance technology, as well as intranasal dermoplasty and estrogen therapy, have been widely employed to control epistaxis [10,11]. Bevacizumab, a monoclonal antibody targeting vascular endothelial growth factor, has also been explored for its potential in treating epistaxis, though further study is required to establish its efficacy [12]. Pazopanib is another VEGF inhibitor that targets the enzyme tyrosine kinase [13]. Despite the wide array of possible treatment options, a definitive “gold standard” for epistaxis management in HHT is yet to be established.

Topical treatments, such as tranexamic acid, selective estrogen receptor modulators (SERMs), propranolol, rose geranium oil, and N-acetylcysteine, have shown potential effectiveness in several preliminary studies [14,15,16,17]. However, their long-term effectiveness and impact on the Epistaxis Severity Score remain unclear.

The Epistaxis Severity Score (ESS) is a gold-standard, patient-reported outcome measure specifically designed to evaluate nosebleed severity in patients with Hereditary Hemorrhagic Telangiectasia (HHT) [18]. It was proposed by Hoag et al. for the International HHT Foundation in 2010 and has been used in various studies to assess the severity and impact of epistaxis in HHT patients [19]. This score is typically documented in patient charts and is confirmed based on documented patient histories [20]. Patients are assigned a score from 1 to 10 based on their answers to six questions—mild (0–4), moderate (4–7), or severe epistaxis (>7) [21,22,23]—with patients having higher ESS scores often requiring closer monitoring and more invasive treatments. Furthermore, the ESS has been shown to have a negative correlation with the physical component score (PCS), indicating that higher severity of nosebleeds can significantly impact the patient’s physical health and quality of life. As a matter of fact, a study from 2015 [24] highlighted the minimal important difference of the ESS in HHT patients, and this threshold is known as the *minimal important difference* (MID). In this study, 604 subjects were recruited, all reporting epistaxis. This study found a significant negative correlation between increasing ESS scores and the Physical Component Summary (PCS), with a correlation coefficient of −0.43 (*p* < 0.001). The minimal important difference (MID) was identified as 0.41 using the anchor-based method and 1.01 with the distribution-based method, resulting in an average MID of 0.71.

Given that a solid consensus on the definitive indications for ablative surgery of nasal telangiectasias has not yet been reached, the choice is usually made jointly between the clinician and the patient. The Policlinico Universitario A Gemelli Center has gained expertise as one of the top three centers in Italy for the surgery of telangiectasias and the post-operative management of HHT patients. Patient adherence to topical treatment is often a factor considered in the decision-making process for potential surgery. Specifically, patients who show poor adherence to topical treatment are directed towards surgery, after a multidisciplinary assessment [25,26].

Through careful clinical observation of patients diagnosed with HHT, we have noticed significant differences in the effectiveness of medical treatments between those who had previously undergone surgical interventions and those who had not. Patients frequently report a worsening of their condition at various times after surgery.

Based on these observations, our study aims to critically reassess the widely held belief that surgical interventions are the optimal approach for treating epistaxis in patients with Hereditary Hemorrhagic Telangiectasia (HHT). For this purpose, we divided our population into two groups: the Surgical Group included patients undergoing invasive or minimally invasive treatments, while the Control Group received no surgical intervention while being assigned a topical therapy instead. Our objective was to evaluate the effectiveness of surgical interventions in reducing the severity of epistaxis in patients with Hereditary Hemorrhagic Telangiectasia. We propose the hypothesis that surgeries and certain interventional procedures, particularly when performed without precise criteria and specific indications, may not only be suboptimal but could also worsen the condition. This study advocates for a more cautious and conservative approach to managing HHT-related epistaxis, emphasizing the need for careful evaluation and selection of treatment strategies [27,28].

## 2. Materials and Methods

Our study included 56 adult patients (aged 18 and above) with a confirmed diagnosis of HHT. The diagnosis is definite if 3 to 4 of the criteria match (recurrent epistaxis, telangiectasias, mainly on the hands, face, and mouth; arteriovenous malformations—AVMs—in major organs; a family history of HHT) with a positive predictive value of 100% [26]. The patients were divided into a Surgical Group (16 females, 14 males; mean age 45.83 ± 16.63), which was part of a follow-up program post-surgical interventions and demonstrated compliance with telemedicine for ongoing evaluation, and a Control Group (13 females, 13 males; mean age 43 ± 16.37), with patients who had never undergone surgery and who were assigned with a topical therapy. The topical therapy assigned to the Control Group patients was structured as follows: nasal washes with saline solution, twice a day; application via a dropper of nasal spray containing cross-linked hyaluronic acid, vitamin A, and vitamin E, 2–3 times a day; followed by the application of a nasal ointment containing hyaluronic acid, twice a day (Rome Italy). The complete list of ingredients and the specific products used in our protocol for the Control Group topical therapy is in Appendix A.

The patients in the Control Group had not undergone any surgery due to refusal by the patient and/or adequate response and control of epistaxis with medical therapy and/or systemic conditions that made it inadvisable to administer general anesthesia to the patient. On the other hand, patients belonging to the Surgical Group had been directed to surgical therapy after adequate consideration of the patient’s compliance with medical therapy, or possible surgical planning in collaboration with other members of the multidisciplinary team or, despite good compliance to the topical therapy, due to the inability to remove nasal packs after a major bleeding episode.

Informed consent was a prerequisite for all participants, ensuring their awareness and agreement with the study’s procedures and objectives.

At the Complex Operational Unit of Ear, Nose and Throat Science of Policlinico Universitario A. Gemelli in Rome, within the framework dedicated to Hereditary Hemorrhagic Telangiectasia, from November 2021 to October 2023, we systematically collected clinical data from 130 patients using the validated ESS questionnaire. Out of these, 44 patients had undergone interventional procedures in their medical history. Only 30 of these patients met the inclusion and exclusion criteria; additionally, a Control Group of 26 subjects with a confirmed diagnosis of HHT was selected and was essentially homogeneous in age and sex to those belonging to the Surgical Group, for whom the ESS was collected at similar times.

Patients undergoing therapy with biological drugs were excluded. This decision was made to eliminate potential variables that could arise from the effects of these medications. Secondly, patients who had undergone procedures but lacked accessible documentation were also excluded. The availability of comprehensive medical records was crucial for accurate assessment and follow-up in this study.

In our study, we observed changes in the ESS over time to evaluate the long-term efficacy of surgical interventions and topical therapies in managing epistaxis in HHT patients. Specifically, the observation times for the ESS included T0, which for surgical patients represented the pre-surgical moment, T1 at 1 month post-surgery, and, finally, T2 at nine months post-surgery. The same ESS collection timelines were maintained for the Control Group, with T0 set as the time before starting the complete topical therapy (nasal washes, nasal spray, and ointments).

The patients of the Surgery Group were stratified into two further groups: Group A (invasive interventional procedures) and Group B (minimally invasive interventional procedures) [16]. The demographics of our population are described in Table 1. For invasive surgical procedures, we refer to coagulation techniques (diode laser, argon plasma) and embolization, and we have included sclerotherapy in this group as well. Minimally invasive procedures encompassed cauterizations with Silver Nitrate (AgNO_3_) and with mono/bipolar tools.

## 3. Results

In our analysis, we aimed to assess the differences in the change of ESS between the Surgical Group and the Control Group at two distinct time intervals: from baseline (T0) to one month (T1), and from baseline to nine months (T2). We conducted statistical analyses to determine whether there were significant differences in age and sex distributions between the Surgical Group (which includes both minimally invasive and invasive surgeries) and the Control Group. The purpose was to ascertain the comparability of these groups in terms of basic demographic characteristics.

### 3.1. Descriptive Analysis

We employed the Chi-square test to assess the differences in sex distribution between the groups. The test yielded a *p*-value of 1.0, indicating no statistically significant difference in sex distribution between the Surgical and Control Groups. This result suggests that both groups were well-matched in terms of gender representation.

Prior to comparing the age distributions, we verified the normality of age data in each group. Both groups demonstrated normally distributed age data, allowing us to use the Student’s *t*-test for independent samples. The *t*-test resulted in a *p*-value of 0.5246. This lack of statistical significance indicates that there were no substantial differences in age distribution between the Surgical and Control Groups.

Prior to statistical comparison, we examined the normality of the distributions of the changes in ESS scores (ΔT1–T0 and ΔT2–T0) for both groups. The Shapiro–Wilk test revealed that the ΔT1–T0 scores for both groups and the ΔT2–T0 scores for the Surgical Group did not follow a normal distribution, while the ΔT2–T0 scores for the Control Group were normally distributed. Given these findings, we elected to use the Mann–Whitney U test, a non-parametric test, for all comparisons to ensure consistency and reliability in the presence of non-normally distributed data.

The differences in ESS scores at baseline between different groups were analyzed. To achieve this, we first ensured that these data met the necessary assumptions for ANOVA. The homogeneity of variances was verified using Levene’s test, which resulted in a *p*-value of 0.222, suggesting that the variance across the groups was homogenous. Subsequently, a one-way ANOVA was conducted to compare the mean ESS scores at T0 among the two groups. The results of the ANOVA indicated no significant differences in the ESS scores at T0 across the groups (F = 1.387, *p* = 0.259). Therefore, based on our analysis, we conclude that there were no statistically significant differences in the ESS scores at T0 among the groups studied.

### 3.2. Statistical Analysis

The results of the Mann–Whitney U test indicated no statistically significant difference between the groups in the short-term change in ESS scores (ΔT1–T0) with a *p*-value of 0.1243. This suggests that both groups experienced similar changes in the severity of epistaxis in the initial four weeks. However, in the long-term comparison (ΔT2–T0), a statistically significant difference was observed (*p* = 0.00016), indicating a disparity in the impact of surgical intervention over a nine-month period. Specifically, the Control Group exhibited a more substantial reduction in ESS scores compared to the Surgical Group (Table 2).

Moreover, our statistical analysis employed repeated measures ANOVA to evaluate the effect of treatment on ESS over the already mentioned three time points: baseline (T0), one month post-treatment (T1), and nine months post-treatment (T2). This analysis was conducted separately for two treatment groups. For the Control Group, the repeated measures ANOVA revealed a statistically significant change in ESS scores over time (F(2, 24) = 8.2144, *p* = 0.0019), indicating that the treatment had a significant impact on ESS scores within this group. For the Surgical Group, the analysis did not show a statistically significant change in ESS scores over time (F(2, 32) = 3.1218, *p* = 0.0577), suggesting that the treatment effect was not statistically significant in this group (Table 3).

The reason why we utilized various statistical approaches to highlight the differences between the Control Group and the Surgical Group is that, given the small sample size compared to other conditions that can ensure a larger number of participants, we wanted to ensure that the results obtained were statistically reliable.

Overall, all these findings highlight the differential impact of the treatment on the two groups. Specifically, the treatment administered to the Control Group significantly improved the ESS scores over the observed periods, suggesting its effectiveness in reducing the severity of epistaxis. In contrast, the treatment effect for the Surgery Group at T2 (nine months) did not reach statistical significance, indicating a lack of substantial improvement in ESS scores in the long term. Indeed, while the surgical interventions had an immediate effect on reducing the severity of epistaxis, this effect was not sustained over a longer period.

The Control Group, which did not undergo surgical treatment, showed a greater improvement in ESS scores over nine months. This outcome raises important considerations about the long-term management of epistaxis in HHT patients and suggests that surgical interventions, while beneficial in the short term, may not provide sustained improvement in comparison to non-surgical management strategies.

The Mann–Whitney U test was employed to compare the efficacy of two surgery treatment groups, categorized as invasive (Group A) and minimally invasive (Group B), in terms of changes in ESS scores. The ESS delta values, calculated as the differences between ESS scores at different time points (T1–T0 and T2–T0), were used as the primary metric for assessing clinical improvement, with lower or more negative deltas indicating greater improvement.

The results of the Mann–Whitney U test for the ESS T1–T0 delta yielded a U statistic of 117.0 and a *p*-value of 0.802, while the test for the ESS T2–T0 delta produced a U statistic of 102.5 with a *p*-value of 0.750. These *p*-values indicate no statistically significant difference in the clinical improvement between the two treatment groups. Consequently, the data suggest that neither treatment method demonstrated a superior outcome in terms of ESS score changes over the observed time periods (Table 4).

## 4. Discussion

Hereditary Hemorrhagic Telangiectasia (HHT) is a rare genetic disorder for which there is currently no definitive cure, with treatment strategies primarily aimed at managing symptoms and enhancing the quality of life for those affected. Over the years, numerous technological advancements have allowed for the exploration of new surgical techniques to manage epistaxis in these patients [29,30]. Being a rare disease, its management is often entrusted to specialized reference centers. These centers are equipped both clinically and surgically to address the complexity and “multifocality” of the clinical manifestations. This specialized approach is crucial for effectively managing the diverse and complex symptoms associated with HHT [31,32].

Even in the context of surgical choices, data emerge on different approaches for managing epistaxis, as suggested in a 2021 study from an Italian center specializing in HHT management [10]. This study highlights that treatment varies with epistaxis severity, favoring less invasive techniques for mild cases and more invasive procedures for severe cases. Endoscopic techniques are emphasized for their reduced morbidity and the benefit of avoiding nasal packing, showcasing the efficacy of these methods in treating HHT-related epistaxis. The Second International Guidelines for the Diagnosis and Management of HHT mention that various ablative therapies can *temporarily* control epistaxis. However, patients should be informed about surgical risks, including perforation of the nasal septum, which, although it can be a possible consequence of repeated bleeding episodes, is more frequently related to the ablative therapies received [33]. Furthermore, it is suggested that clinicians consider the use of systemic antiangiogenic medications for the treatment of epistaxis that has not been effectively managed by moisturizing topical therapies, ablative procedures, or tranexamic acid, highlighting a comprehensive approach to care [25].

Based on our experience, topical therapies using nasal sprays and ointments containing hyaluronic acid and other substances with primarily moisturizing and barrier function can represent an excellent long-term option for HHT patients suffering from epistaxis. Several studies suggest a shift in the management of epistaxis in HHT from primarily surgical interventions towards incorporating systemic therapies [6,34]. These studies highlight that, while traditional treatment recommendations focused on surgical options, the Second International HHT Guidelines now place systemic therapies, such as oral tranexamic acid and systemic antiangiogenic agents (bevacizumab and thalidomide), on equal standing with local surgical treatments for epistaxis management. This evolution in treatment approach reflects the development of new systemic targeted therapies addressing the underlying pathophysiology of HHT and is based on evidence demonstrating the efficacy of these systemic treatments in reducing epistaxis, offering a broader range of options for patients unresponsive to moisturizing topical therapies alone.

In our study, our primary objective was to evaluate the long-term efficacy of the therapeutic approaches for managing epistaxis in patients with HHT. We focused on comparing the outcomes between a Surgical Group and a Control Group over two specific timeframes: one month (T1) and nine months (T2) post-therapy. The analysis of changes in ESS from baseline (T0) to these time points provided insights into the short-term and long-term effectiveness of the treatments. Through a comparative analysis of long-term outcomes between patients who underwent surgical treatments and those who received targeted topical therapies, our research was intended to highlight the possible adverse effects of surgical interventions.

For the Control Group, the treatment regimen consisted of conservative management strategies frequently utilized at our center. These included nasal washes with saline solutions, the application of nasal sprays, mainly administered as drops (composed of hyaluronic acid), and the use of nasal ointments following episodes of epistaxis.

Our statistical analysis revealed no significant differences between the surgical and Control Groups in the short-term (T1) change in ESS (Δ T1–T0), as indicated by a Mann–Whitney U test *p*-value of 0.1243. However, a significant difference emerged in the long-term (T2) analysis (Δ T2–T0) with a *p*-value of 0.00016, suggesting a disparity in the impact of the surgical intervention over a nine-month period. Interestingly, while the Surgical Group exhibited immediate benefits, indicated by the changes in ESS at T1, these benefits were not sustained at T2. The Control Group, which adhered to conservative management, showed greater improvement in ESS scores at the nine-month mark. This observation underscores that, although surgical interventions provide immediate relief from epistaxis, their benefits might diminish over time, potentially resulting in conditions worse than the baseline.

Within the Surgical Group, regarding the two types of treatment (minimally invasive and invasive), we have not identified a statistically significant difference in terms of long-term reduction of the ESS; this supports our hypothesis that the nasal mucosa of patients with HHT is differently predisposed to respond to structural alterations induced by surgical treatments compared to individuals not suffering from HHT. We believe, based on our personal experience, that the extent of the surgery performed does not determine the outcome. Further studies on the different morphology and characteristics of nasal telangiectasias are needed to confirm this hypothesis. An interesting perspective could be to conduct a randomized clinical trial on the evaluation of surgical outcomes for different types of surgery, based on the grading of nasal telangiectasias detected pre-operatively and to correlate these results with the patient’s genetic makeup.

### Limitations

Although the sample size in this study is not extensive in numerical terms, it is important to consider the strict inclusion criteria implemented to mitigate potential selection bias, especially given that Rendu–Osler–Weber syndrome (HHT) is a rare disease with limited case numbers compared to other otolaryngological conditions.

This study indicates that surgical treatments initially provide short-term benefits (one month post-treatment); however, these advantages appear to diminish over time (nine months post-treatment). On the other hand, conservative treatments like nasal washes, sprays, and ointments show consistent improvement in managing epistaxis over this period, suggesting they may offer more enduring benefits than surgery.

Another limitation of the current study is the lack of complete randomization in assigning patients to the two groups (Control Group and Surgery Group). However, we can clarify that not only patients with more severe symptoms were directed to surgery, and this is supported by the lack of statistically significant differences in the ESS scores at T0 between the Control Group and the Surgery Group. As a matter of fact, Surgery Group also included patients who had undergone surgical therapy due to poor compliance with the prescribed topical therapy, or patients who were directed to surgery based on medical needs identified collectively by the multidisciplinary team; for patients who underwent nasal packing at another hospital during an episode of epistaxis and were subsequently directed to our center for the removal of the packs, surgical intervention was selected in cases where attempts to remove the packs were unsuccessful.

The notion that surgery should primarily be considered for severe cases refractory to other treatments opens opportunities for advancing research in medical management strategies for HHT. This perspective encourages a thorough evaluation of current medical treatments and the exploration of new, more precise therapies that tackle HHT’s root causes with minimal adverse effects. It underscores the necessity of prompt, accurate diagnoses, continuous patient monitoring, and flexible treatment approaches that can be adjusted based on disease evolution or individual responses to therapy.

## 5. Conclusions

Our research indicates that for HHT patients, surgical interventions should generally be seen as a last resort, particularly when other medical treatments fail to yield results. The evidence suggests a preference for a conservative approach to managing epistaxis, emphasizing the importance of preserving tissue and minimizing interventions. When surgery is deemed necessary, it should aim to address specific issues, such as treating larger or more problematic telangiectasias, and should proceed with utmost care to avoid damaging the nasal mucosa.

The necessity for future research involving larger and more varied participant groups is highlighted to reinforce these conclusions and aid in establishing thorough treatment guidelines for HHT. The effectiveness of interventional procedures appears limited, with a tendency to potentially exacerbate conditions over time. The establishment of an international, or at least a European, registry for this rare condition is advocated to facilitate the evaluation of broader patient data and the formulation of updated, effective international guidelines for epistaxis treatment in HHT.

## Figures and Tables

**Table 1:** Demographics for the selected groups.

**Sex Distribution**			
	**Control Group**	**Surgery Group A**	**Surgery Group B**
Total Females	13	9	7
Total Males	13	8	6
**Age Distribution**			
	**Control Group**	**Surgery Group A**	**Surgery Group B**
Total Patients	25	17	13
Mean Age	43	50.94	39.15
Age Std Dev	16.37	15.86	15.74

**Table 2:** Mann–Whitney Test for differences between Surgical Group and Control Group in terms of ESS at different times (ESS T1–T0 and ESS T2–T0). Dif T1-T0 indicates the difference between the mean ESS at T1 and T0; Dif T2–T0 indicates the mean difference between ESS at T2 and T0.

Group	ESS (Mean ± SD)		*p* Value (Mann–Whitney U Test)
Surgical Group	T0	5.21 ± 2.59	
	T1	3.39 ± 2.09	
	T2	5.14 ± 2.42	
Control Group	T0	4.67 ± 1.79	
	T1	3.50 ± 0.98	
	T2	2.78 ± 1.05	
Surgical Group	Dif. T1–T0	−1.17 ± 1.37	0.1243
Control Group	Dif T1–T0	−1.83 ± 3.36	
Surgical Group	Dif T2–T0	−0.08 ± 3.08	0.00016
Control Group	Dif T2–T0	−1.89± 1.51	

**Table 3:** Results of the repeated measures ANOVA to evaluate the effect of treatment on ESS over the three time points (T0–T1–T2).

	F Value	Num DF	Den DF	*p*-Value
Control Group	8.2144	2	24	0.0019
Surgery Group	3.1218	2	32	0.0577

**Table 4:** Mann–Whitney Test for differences between Group A and B, showing no statistical difference between the two treatments (mini-invasive and invasive surgery).

Comparison	Mann–Whitney U Statistic	*p*-Value
ΔESS T1–T0	117	0.8015
ΔESS T2–T0	102.5	0.7502

## Data Availability

The datasets presented in this article are not readily available because the data are part of an ongoing study. Requests to access the datasets should be directed to giuliocesare.passali@unicatt.it.

## Appendix A

Nasal spray composition: D-panthenol, vitamin E acetate, vitamin A palmitate, biotin, hydrogenated castor oil 40 o.e., crosslinked hyaluronic acid, disodium EDTA, sodium hydroxymethylglycinate, potassium sorbate, natural flavor, isotonic buffered solution at pH 7.2; EU patent (medical device).

Nasal ointment composition: hyaluronic acid sodium salt, Centella asiatica, calendula, aloe vera, sweet almond oil, BHT, vitamin E acetate, propylene glycol, white petrolatum, petrolatum oil, castor oil, carnauba wax, benzalkonium chloride.
